# Telepathology and Optical Biopsy

**DOI:** 10.1155/2009/740712

**Published:** 2010-03-18

**Authors:** Olga Ferrer-Roca

**Affiliations:** Faculty of Medicine, University of La Laguna, 38071 Canary Islands, Spain

## Abstract

The ability to obtain information about the structure of tissue without taking a sample for pathology has opened the way for new diagnostic techniques. The present paper reviews all currently available techniques capable of producing an optical biopsy, with or without morphological images. Most of these techniques are carried out by physicians who are not specialized in pathology and therefore not trained to interpret the results as a pathologist would. In these cases, the use of telepathology or distant consultation techniques is essential.

## 1. Introduction

An optical biopsy (OB) [1] is the examination of tissue using a nonintrusive diagnostic method. It is performed with one of the following techniques: laser, optical coherence tomography (OCT), infrared, fluorescence, spectroscopy, and so forth. This means that it is not necessary to extract tissue to make a diagnosis. Tissue is accessed through the skin or by endoscopy. 

In pathology the “gold standard” is the histology of the fixed tissue (dead tissue). In OB techniques, images are obtained in real time together with complementary information, which allows evaluation of the disease in vivo, but gold standards are still lacking. 

**Figure 1:**
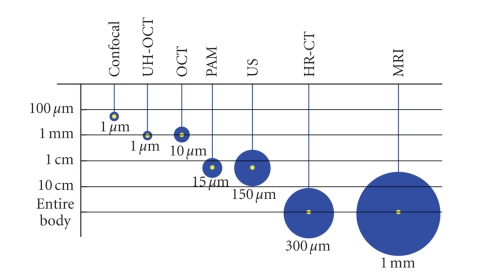
Spatial resolution (circles) and axial resolution (*Y*-axis) of several medical imaging techniques. Light methods are 1-2 orders of magnitude higher in spatial resolution. (HR-CT: high resolution computer tomography, MRI: Magnetic resonance imaging, the rest in the text.).

From the technical point of view, the methods can be divided in two major groups: those based on morphological images (Figure 1) and those not associated with images. 

The term Optical Biopsy was established for the latter group, but if we use the definition *use of optical energy to obtain information regarding structure and function of the tissue, without disrupting it*, then this includes any of the noninvasive techniques. This definition is closer to pathology competencies by training [2] because the diagnosis is based either on normal tissue or on changes in cell or tissue morphology.

## 2. Super Resolution

**Figure 2:**
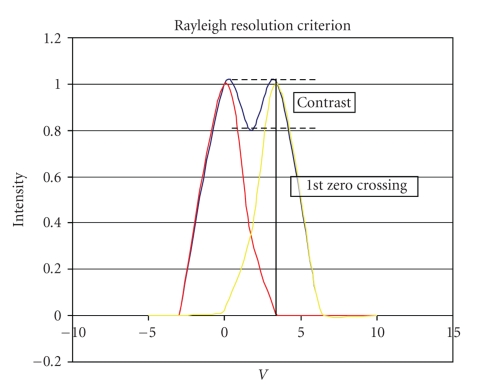
According to the Rayleigh law, two objects are visible if they are located at 0.61 *λ*/NA distance and have a contrast of 26%, because lateral resolution depends on luminosity and contrast of the object.

In general, OB images have higher resolution than the *Rayleigh Law *0.61*λ/NA which considers that two points can be resolved when the centre of the *point spread function* (PSF) is inside the *first zero* of the PSF centred in the second point, provided that contrast is higher than 26%, because lateral resolution depends on object luminance and contrast (Figure 2).

The **superesolution formula (** **N** **)** is such that *λ*/2 ·**N**· NA is the limit of resolution with N*resolution being equal to *λ*/2 · NA. The optical resolution is limited by sampling in CCD cameras. According to *Nyquist sampling theory*, to distinguish an object this should be separated at least by 2.3 to 3 times the microscopic power (*M*) per the optical resolution (Δ*d*) divided by the size of the camera pixel (Δ*x*)

(1) $$\Delta d*\frac{M}{\Delta x}=\;\text{2.3–3.}$$

**Figure 3:**
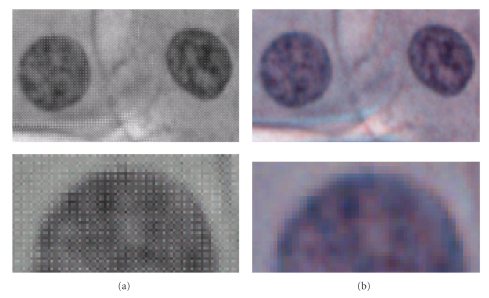
Optical system 15x NA = 0.75, taken with a CCD of 2.7 *μ*m pixels. The optical resolution of 0.45 *μ*m is sampled with 2.5 pixels, insufficient considering the 4 pixels colour integration. Raw images (a) and their RGB transformation (b). In the raw image the 2G-R-B mosaic of the single-colour-chip is visible.

Furthermore, pixel size (the smallest is 2.7 *μ*m in 2/3^′′^ chips [3]), as well as the degree of integration of sensitive RGB pixels in single chip colour cameras (see Figure 3), also modifies the resolution. 

Super resolution can be achieved using **information theory** tools, such as image analysis and pattern recognition. Starting with *noise control* of the image capture device by averaging, followed by *adaptive contrast filters*, which detect borders and operate inside the region, up to the *Trough focus scanning optical microscope* (TSOM) [4] technique that achieves 3 nm of resolution. 

Numerous **microscopic techniques** based on structured illumination, interferometry, and holography also achieve super resolution; while **spectroscopic techniques** that detect physical and chemical specimen properties (**spectral pathology**) can also be at the super resolution limit. 

Finally, **optic nonlinear techniques** use excitation coherent light whose tissue scattering is not related to the incident light (double frequency) providing information about structure and molecular interactions. These systems are called **chemical microscopes** because they detect molecular changes or molecular arrangement and distribution. 

All of them can be miniaturized using new materials and micro-electronic machines (MEMs) and incorporated into endoscopic devices to obtain **chemical or imaging micro-endoscopies**; these are usually managed by endoscopists not trained in the pathology domain. This enhances not only the *Push Endoscopy* (PE) but *Double balloon endoscopy* (DBE) that allows visualising the whole small intestine up to the more recent *Video-endoscopy capsule* (VCE), a wireless endoscopic capsule (WCE: *wireless endoscopic capsule*). Any of them can carry optical biopsy systems.

## 3. Techniques

The most popular super resolution systems, in particular fluorescence, use* structured illumination*. Confocal microscopy uses a light beam at the limit of optical diffraction, and other technique such as two photon imaging or third harmonics use smaller light beams. 

*Interference fringe* projection in the object is another method. Finally the most sophisticated applications correlate captured and expected images as in *holographic techniques* and *image processing*; when both images are similar, differences reach super resolution. 

### 3.1. Structured Illumination

Excited molecules tend to return to their initial level of vibration. If emitted photons are of similar energy and wavelength as incident light we get a Rayleigh scatter. Fraction emitting photons different from initial excitation produce a Raman scatter: predominantly of higher energy producing the “*Stokes*” effect, but also of lower energy in the “*antistokes*” effect. 

#### 3.1.1. Fluorescence Spectroscopy

Fluorescence spectroscopy studies properties of tissue taking into consideration the intensity and wavelength emitted by fluorescent or autofluorescent objects excited by UV, near infrared-NIR (640–850 nm) or compact/ tuned *Laser induced fluorescence* (LIF). 

There are two methods: *Light scattering spectroscopy* (LSS) for macroscopic areas and *Fast excitation emission matrix* (FastEEM) for microscopic areas using optical fibers. Three types of information can be studied, together known as *tri-model spectroscopy* (TMS): fluorescence, reflectance and elastic dispersion of light.

**(a) LSS**

LLS does not require structured illumination. Systems can be either “home-made” or commercial devices. This is the case with *visual enhanced lesion scope* (**VELscope**) from LED Dental Inc., used in the oral cavity. (**PMTI**)* Photonic Molecular Tissue Imager* from Mediscience Technologies uses UV-320 nm and yellow light at 580 nm in cancer detection (CD-ratiometer) and human papilloma virus (HPV). *Cervical imaging system* (**LUMA**) from MediSpectra Inc., acquired by SprectraScience uses UV and visible light between 360 and 720 nm. *HyperSpectral Diagnostic Imaging system* (**HSDI**) from STI Medical systems, Hawaii, registered the term “Virtual Biopsy” as capture and automatic analysis by CAD (*computer-aid-diagnosis*) of direct, fluorescent, and scattered white light, to localize malignancy risk areas. Simpler is *Dynamic Spectral Imaging System* (**DySIS**) from Forth-photonics showing pseudocolor after cleaning the colposcopic area with dilute acetic acid [5].

Finally *light-induced fluorescence endoscope* (**OncoLIFE**) from Xillix uses blue light (425–455 nm) with a xenon lamp. Fluorescence is collected with a CCD and analyzed with green (480–580 nm) and red filter (620–720 nm). This technique has been incorporated into gastrointestinal tract analysis with *Fujii intelligent chromoendoscopy* (FICE) technology from **Fujinon**.

**(b) FastEEM [see [6]]**

This method has structured illumination: fluorescence is excited by a pulse laser (308–480 nm) and two pulses of white light through an optical fibre probe (270–800 nm). Light is collected through a diffraction grating and sent to a CCD intensifier. A spectrograph analyzes the first-order diffraction and eliminates the second order (>540 nm) with a high-pass filter.

**WavSTAT **[7] system developed by SpectraScience uses tuned LIF at 410 nm. The optical fibre LIF probe can be integrated in an MIS-colposcope (*Multispectral Imaging system*) or in an endoscope through the biopsy channel.

All systems allow “cervicograms-tissuegrams,” which means that MIS-colposcopy and tissue images that can be stored and consulted at distance. In fact it is a virtual biopsy if the term had not been registered by STI Medical Systems.

#### 3.1.2. FTIR Spectroscopy

*Fourier transform Infrared spectroscopy* (FTIR) similarly to any IR system is a process of absorption but in this case analyzed in the frequency domain. 

Fluorescence and the FTIR are the most sensitive and popular methods of optical biopsy. In cancer of the uterine cervix, the FTIR has a sensitivity of 98.6% and a specificity of 98.8%, with positive and negative predictive values of 99.5% and 96.5%, respectively, while PAP-cytology achieves 95.9% and 72.3%.

#### 3.1.3. Raman Spectroscopy

Raman spectroscopy includes: resonance, *surface-enhanced Raman scattering* (SERS), and microspectroscopy. 

Raman phenomena occur with any type of incident light, but probes have difficulties detecting spatial distribution of the tissue, because the signal is very low. In medicine, Raman spectroscopy in near infrared **(Raman-NIR)** is used to detect preneoplasic skin lesions, gastrointestinal lesions by endoscopy (see below VCE), changes linked to atheromatosis, as well as a therapeutic aid for detecting tumour margins in surgery. It has also been used for noninvasive analysis of blood or drugs. 

A structured illumination is obtained with the **Raman-** *hollow optical fiber* (**HOF**) whose optical fibre does not produce noise because the light is limited to the centre. The probes do not require filters, have several wavelengths, and one fibre can conduct the excitation light and collect scatter light; the resulting Raman scatter has a high *signal to noise ratio* (SNR) and does not require background correction.

#### 3.1.4. Raman Microscopy

Raman spectroscopy has been integrated into regular microscopy as well as confocal microscopy. In the latter, 3D images of high resolution are obtained to study tissue properties at a subcellular level, being of great value in **chemical microscopy**. 

#### 3.1.5. Confocal Microscopy

Based on structured illumination, it uses two small light apertures (pinholes) in conjugated planes (confocal); one in front of the excitation light and the other in front of the detector. Thus, fluorescence out of focus is avoided since the confocal aperture only allows light from the focal plane, improving lateral, and axial resolution. 

Axial resolution allows optical sectioning, focussing one plane at a time and reconstructing images electronically. 

Confocal laser system miniaturization has led to **endomicroscopy** or microscopy with **confocal endoscopes. **They use ultrashort pulsed lasers with rod GRIN microlenses (*gradient refractile index*) to control chromatic dispersion and phase modulation. Initially these were of 0.9 mm in diameter, with 30 Kfibers directly connected to a GRIN (1 mm. diameter) of 4.8 magnification (low NA = 0.2), with a total diameter of 1.2 mm; the space between fibers was 3 *μ*m limiting the lateral resolution of sampled area to 0.6 *μ*m [8]. 

**Figure 4:**
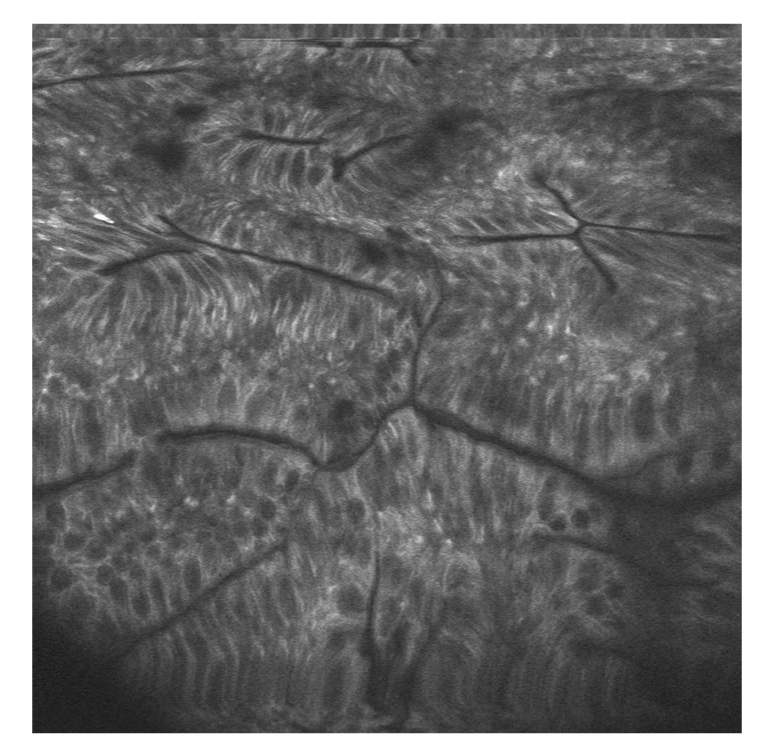
Confocal Endomicroscopy. Hyperplasic Polyp. Image provided by Priv.-Doz. Dr. med. Martin Götz, Oberarzt Gastroenterologie I, Medizinische Klinik und Poliklinik, Universitätsklinik Mainz.

The most widely used system is **Optiscan** (Optiscan, Australia) [9] integrated on the tip of a conventional colonoscope (Pentax EC3870K; Pentax, Tokyo, Japan), which allows an optical biopsy of the epithelial surface and *lamina propia *during endoscopy (Figure 4). Contrast enhanced with sodium fluoresceine or methylene blue it gives rise to multispectral confocal microendoscopy (MIS) capable of detecting immune markers such as anti-CD44V6 for aberrant crypts and producing an **immune-microendoscopy **[10]. 

Mauna Kea Technologies has built **Cellvizio**, a family of confocal-microscopy probes on miniaturized objectives compatible with any colonoscope, gastroscope, or bronchoscope that allows reaching very small conducts obtaining cholangioscopies and alveoloscopies. Their lateral resolution is 1 *μ*m and the axial resolution is 150 *μ*m. Their diameters are from 0.93 to 2.5 mm and they are up to 4 meters long.

#### 3.1.6. Endoscopic Capsule and OB

*Video-endoscopy capsule* (VCE) allows direct examination of the small intestine in a noninvasive, secure and well-tolerated manner, although stenosis and diverticulus are contraindicated. For that reason the capsules are now marked with RFID to easily locate and extract them. Several types are currently available in [11–14]. 

WEC or wireless endoscopic capsule **M2A** (PillCam SB from Given Imaging) is of 3.7 g, and measures 11 × 26 mm, with a CMOS camera and 6 LEDs, equipped with UHF transmission, sending 2 frames per second during 7 hours. 

The **EC type 1** is from Olympus with a high resolution CCD-camera and a real-time viewer. 

The **CPE**—Compact Photonic Explorer, from Mediscience Technology, contains spectroscopic fluorescence able to detect malignant lesions even at the molecular level. 

The third generation of capsules is the **Norika 3 RF Endoscopic Robot Capsule **in 2001. It does not have batteries because it is charged with an external electromagnetic field that also powers rotation. It measures 9 × 23 mm and 40% is empty and available for surgical or therapeutic gadgets such as sprays, lasers, and pH detectors. New releases are of 5.8 × 15 mm which can be used in children. They use the mosaic technique to combine images and provide a panoramic view. 

**Figure 5:**
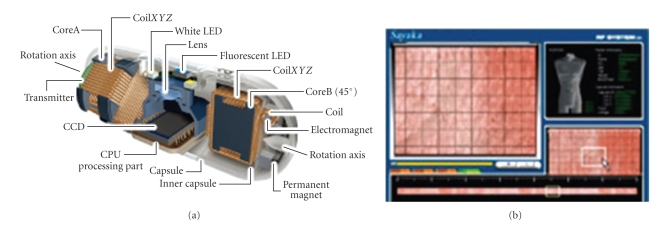
Sayaka CE. Vision is lateral; therefore snapshots are taken while rotating and are composed in mosaic. It is from http://www.rfamerica.com/sayaka/.

**Sayaka-CE** from RF system Lab, Japan, sends 30 fps, does not use batteries, and also builds mosaics (Figure 5). 

The fourth generation incorporates active motion and a miniaturized sampling system. This is the case with **Versatile** (*Endoscopic Capsule for gastrointestinal Tumor Recognition*) that also includes NIR-spectroscopy.

### 3.2. Interferometry

Resolution improvement with *differential interference contrast* (DIC), the Nomarski technique, has been known for many years. 

Today, the interferometric techniques with diffraction gate can be compared to confocal microscopy but with better tissue penetration (Figure 1); axial resolution is linked to the coherence of light and transversal or lateral resolution to the size of the light beam. 

There are three modalities with low coherence light or *Low coherence interferometry* (LCI); *coherence probe microscopy* (CPM), *optical coherence tomography* (OCT), and *optical coherence microscopy* (OCM). The first uses halogen light and the other two super-LEDs or (SDL) super-luminescence laser diodes. The applications with halogen light are known as full field (FF-OCT and FF-OCM).

#### 3.2.1. Imaging Interferometric Microscopy (IIM)

IIM [15] can be adapted to existing microscopes without entering the pupil plane of the objective. It uses structured light obtained through a gate of less than wavelength (*λ*), at the limit of the NA of the optical system, which produces a shift in spatial frequencies, recording and combining several images in amplitude and phase to improve the resolution. 

IIM works at lower power with low aperture objectives (NA <0.5) but obtains twice the resolution of high aperture objectives (NA = 1.4). The depth of field (DoF), vision area and working distance, is that of a low power objective, although the final integrated image has a resolution at the linear limit of the transmission media (refraction index *n*) *λ*/4*n*. By shifting the objective plane and transforming the observed spatial frequencies (reference image) into spatial frequencies of the real image, the resolution extends to the bandwidth limit of the transmission media *λ*/4.

Molecular interferometry is a step forward; MI2 or *Molecular Interferometric Imaging* allows the detection of biomolecules.

#### 3.2.2. Spectral Single Fibre Interferometry

Endoscopes with high quality image are large because the sensors are also large (1 cm or more in diameter). Standard microendoscopes (2.4 to 1.2 mm in diameter) use a limited bundle of optical fibres, improving flexibility but losing image quality. Single-fibre microendoscopes, with the thickness of a hair (80 nm) in 2006, have HOF effect, and are made of *photonic crystal fibers (PCF*). 

SEE or *spectrally encoded endoscopy* uses multicolour light projected into the tissues through a single fibre of the thickness of a hair. The scattering is collected and the various wavelengths are analyzed by spectroscopy. Simultaneously, an interferometer computes the structural information based on two light sources giving a 3D image. The result is a FastEEM on a 3D image.

#### 3.2.3. Optical Coherence Tomography

OCT is a noninvasive technique that uses low coherence light sources such as SLD, some of them in the NIR-region (NIRIS OCT^TM^ of Imalux), to produce tomography by interferometry TDOCT-*time-domain OCT* or interferometry SDOCT-*spectral-domain OCT*. 

**Figure 6:**
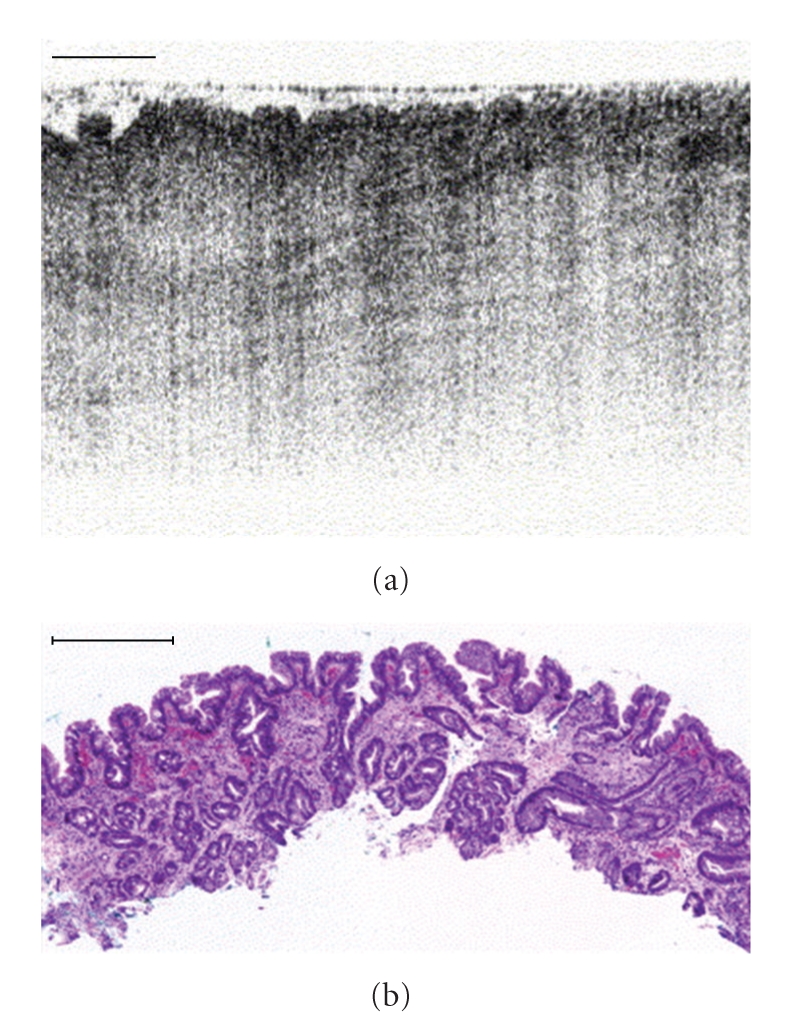
Endoscopy of UH-OCT. The upper image is the OB and the lower image shows the histology of the area. (Taken from P. Hsiung, L. Pantanowitz, et al. (2005)   http://www.med.upenn.edu/bmbgrad/Program/course_descriptions/BMB_620/10212005_chen.pdf.)

By definition it works with low numerical apertures (NA <0.5) and has good penetration (1–3 mm). In 1991, the axial and lateral resolution using SLDs was of 30 *μ*m; in 2001, using broadband (range > 100 nm) pulsed-lasers it was of 10 *μ*m (H-OCT or *high resolution-OCT*), and today using Ultra-high resolution (UH-OCT) it reaches 0.5 *μ*m (Figure 6). 

OCT is widely used in ophthalmology, and is extending into endoscopy (i.e., Bladder tumor detection with a sensitivity of 92% and a specificity of 85%), colonoscopy and other imagining techniques. There are on the market **OCT-endoscopes** of 1 mm in diameter and **OCT-fiberscopes** of 400 *μ*m in diameter with GRIN or drum optics [16–19]. 

In ophthalmology, adaptive optics (AOs) is required to compensate for irregularities of the ocular system, which makes the devices much more expensive [20, 21]. 

#### 3.2.4. Optical Coherence Microscopy

Although OCM is used when the numeric aperture is high (NA >0.5) the fact is that there are GRINs optics with NA = 1.2, achieving important optical detail. Furthermore, on a classical OCT a synthetic aperture can be built to yield an *Interferometric Synthetic Aperture Microscopy* (ISAM) [22], whose advantages are used in *Optical frequency domain imaging* (OFDI). It is well known that the depth of field (DoF) and resolution are inversely related, because one improves when the other decreases and vice versa. With the ISAM method there is no such limitation because it captures stable phase data including reference phase (i.e., coverslip) in the object. The process consists of a series of filters and mapping in the Fourier domain. The result produces high quality histological detail. 

#### 3.2.5. Full-Field Optical Coherence

The macroscopic OCT is the FFOCT or *full-field optical coherence tomography* that uses simple halogen lamps and CCD cameras as detectors, achieving an SNR of 90–100 dB. 

Similarly, FF-OCM or *Full-Field Optical Coherence Microscopy *[23] uses broadband thermal light from a tungsten lamp to produce OCM spectroscopy. This technique, with low power water immersion, objectives 10x of low numeric aperture NA = 0.3, captures spectral information with colour cameras, and produces high resolution images (axial 0.8 *μ*m and lateral 1.4 *μ*m) by means of image processing. 

Microscopic detail provides a pseudohistological stain in the *hue-saturation-luminescence* (HSL) color space, where *H* represents *λ* variation, *S* temporal shift, and *L* is constant [24]. In other words, hue is the predominant wavelength, saturating the spectral purity and luminancing the colour intensity. In the *H*-channel, changes in the wavelength of the reflected light are codified in red, green, or yellow depending on whether they increase, decrease, or are maintained. In the *S*-channel of each wavelength, the reflectivity of the tissue is stored while *L*-channel is constant [25]. 

#### 3.2.6. Optical Frequency Domain Imaging

OFDI is an improvement on the OCT, because instead of examining point by point, 1000 points are analyzed simultaneously. The tip of the probe of the optical fibre is permanently rotating and emits a laser light that changes the wavelength from 1264 to 1376 nm. An image is created measuring the temporal delay of the echo as well as the spectral interference between the tissue and the reference in each wavelength, whose Fourier transformation (optical frequency domain) gives a microscopic view.

#### 3.2.7. Terahertz Interferometer Imaging

This uses electromagnetic high wavelength (THz), localized between microwaves (MW) and infrared (IR) light, with a frequency of 10^12^ Hz. These waves propagate in metals and nonpolar material and are therefore highly appreciated as metal detectors for weapons and so forth. They are highly sensitive to molecular resonance and therefore used in interferometric image sensors and detectors. The THz generators are classified in three categories: thermal incoherent, broadband pulsed, and continuous wave narrow band.

The majority use **TDT** *or pulsed spectroscopic imaging based on time-domain terahertz spectroscopy*. It allows one to obtain any type of information regarding spectral absorption, depth, and type of objects. The disadvantage is that the optical components are large, expensive, and with low potential. 

In contrast, **CW** or *continuous-wave THz imaging system* is simple, of low cost, and does not require expensive devices or complex optics. For a multifunction broadband system we can use multiple discrete frequencies or tuned lasers. Both phase and amplitude can be obtained by interferometry. 

**THz-endoscopes** on *subwavelength plastic fiber *are easy to implement. Reflection 3D-THz images have a reasonable SNR and high spatial resolution. It is a matter of time before we obtain a quicker detector to improve endoscopic molecular imaging.

### 3.3. Holographic Techniques

In holography, the dispersion of light from an object is recorded in a medium while it is illuminated with a second point of light (reference light). The light field obtained from the interference of both light sources is the hologram of the object.

#### 3.3.1. Digital Holographic Imaging (DHI)

Holographic gratings are used in numerous noninvasive applications of UV or medium-IR. Only those of industrial quality reach the required SNR. These products can be customized to a specific wavelength.

#### 3.3.2. Digital Holographic Microscopy (DHM)

**Single mode holographic endoscopes **produce a 3D image of high DoF. They consist of a miniaturized holographic system capable of recording reflection holograms, with 3 parts: the cartridge, the diagram, and a single mode fiber of 4 *μ*m in diameter. It has an NA that allows a lateral resolution of 7 *μ*m.

Resulting holograms are visualized in potent microscopes to see individual cells. Contrast methods (fluorescence, staining) are often used before recording, as in gynaecology or enteroscopy.

#### 3.3.3. Quantum Holography

With the new techniques in holography we can obtain a “**molecular hologram**” using incident electrons. The result is an electronic object in 3D that can be seen, for example, in scanning tunnelling microscopes. In the same hologram we can record several images in several wavelengths that can be read as a book just by changing the wavelength. This information can be miniaturized to an extreme to finally get a quantum holograph.

### 3.4. Nonlinear Illumination

Directly related with laser technology, it uses ultrashort pulses (high peak and low potency) on optical fibers with high aperture lenses. Specimen response is nonlinear to the incident light, but parametric since there is no energy transfer. Two phenomena are included: Harmonic generation and surface enhanced Raman scattering (SERS).

**Nonlinear single fiber optical endoscopes** of low numeric aperture use thick optical fibers with limited sensitivity. To improve them, long and thin fibers are used instead, very efficient in both senses (excitation collection and emission) emitting light at the limit of optical diffraction and capable of supporting laser ultrashort pulses. These are the PCFs *photonic crystal fibers* that together with MEMs allow building miniaturized single-fiber endoscopes.

These techniques will only be covered superficially since they integrate **chemical microscopes** with nanometric resolution far removed from surgical pathology. 

#### 3.4.1. Coherent Antistokes Raman Scattering (CARS)

CARS microscope consists of two lasers (pump and antiStoke) synchronized in time, and whose frequency depends on the particle under study. CARS includes resonance and nonresonance. The first is proportional to the square of Raman scatter, and the resulting signal is the cube of the initial one. Therefore, this is a nonlinear technique that improves spontaneous Raman scattering. It is very sensitive to Carbon-Hydrogen vibration and for that reason is used to detect lipids. 

#### 3.4.2. CARS Confocal Microscopy

CARS [26] has been integrated with confocal microendoscopy in order to obtain high resolution images of vibrating particles. Confocality is coherent with CARS. These endoscopes are single fiber, with MEMs and NIR.

#### 3.4.3. Nonlinear Interferometric Vibrational Imaging (NIVI)

Even better is the NIVI technique. It uses the principles of CARS and OCM to obtain a transversal section of the particular molecules inside the specimen. Two CARS signals are generated, one from the reference molecules and the other from the molecule we wish to study. The two coherent light signals mutually interfere; the *envelope *or combined signal measures the concentration and the *interference fringes* give information regarding the phase and allow us to determine the vibrational characteristics of the molecules. 

#### 3.4.4. Nonlinear Deep Ultra-Violet Microscopy (DUV)

DUV microscopy on thick tissues extends regular optical microscopy to the region of the DUV in order to detect endogenous signals from nonmarked materials. DUV wavelength is between 230 nm and 350 nm, although it is not completely quantified, because it cannot be transmitted through glass and therefore cannot be observed with usual methodology. 

#### 3.4.5. Photon Excitation Microscopy

We can use a single photon *one-photon excitation* (OPE) or two photons TPE (*Two-photon excitation microscopy*), the latter with IR at 730 nm. The IR photons improve tissue penetration due to the fact that, in contrast to UV, the IR has less absorption coefficient and a limited dispersion coefficient. Finally, multiphoton excitation MPE uses the same principle but with a pulsed laser of 100 fs at 80 MHz and wavelength from 700 to 1050 nm [27]. Since fluorescence only appears in the focused volume there is no excitation out of this and the 3D resolution is intrinsic. 

**Photon excitation endoscopes **had been miniaturized over TPE with MEMs scanners and ultrashort pulsed-lasers on *hollow-core photonic-bandgap fiber* (HOF) that virtually eliminates pulse distorsion, which is currently applied in neurobiology. 

#### 3.4.6. Second Harmonic Generation Micrcoscope

SHG microscopes detect the second harmonic which is a property of noncentrosymetric structures such as collagen and has been applied with success for the study of the cornea, and lamina cribosa of the sclera.

#### 3.4.7. Near Field Scanning Optical Microscope

The NSOM scans the object with a wave of light under the wavelength *a* ≪ *λ*. The emission point is located close to the object (distance <*a*), obtaining a resolution closer to *a* and an **N** = *λ*/2*a* 10 times superior. 

The optical fiber is usually tapped and the light reflects on the tapper, with only a very small wavelength leaving the fiber. 

It is often combined with other photonic excitation techniques such as the trifunctional microscope in which one can move from CM or confocal to SNOM and to AFM (atomic force microscope), moving the microscopic tower.

#### 3.4.8. Scattering-SNOM

Another nanometric solution is the s-*scattering-type Scanning Near-field Optical Microscopy* (SNOM). It uses AFM to concentrate the light on one point. This allows 20 nm resolution, breaking the diffraction barrier with visible light, IR and THz.

#### 3.4.9. Atomic Force Microscope

AFM allows nanometric resolution. It works following the surface of the object. The sensitive point is located at the end of the cantilever and can work touching the surface of the specimen or not.

### 3.5. Hybrid Methods

#### 3.5.1. Photo Acoustic Microscopy (PAM)

This is a hybrid method since it includes three techniques: optic irradiation, ultrasound detection, and image production. Illumination is performed with 804 nm wavelength that produces a deformation in the tissue which is captured by a 5 MHz Ultrasound probe, providing high resolution images (axial resolution of 15 *μ*m and lateral resolution of 45 *μ*m) with high penetration in the tissue (up to 3 cm) [28, 29]. 

#### 3.5.2. Through-Focus Scanning-Optical-Microscope (TSOM) Imaging

Regular microscopes cannot normally be used for nanometric detection (<100 nm) because of optical resolution limitations due to the wavelength used. Nevertheless, the TSOM [4] method is able to overcome these limitations.

The method uses noncoherent, nonstructured illumination processed by image analysis of images taken by a regular camera through a regular optical microscope equipped with automatic staging and established *Z*-focus movements. This method extends the range of the regular optical microscope to the nanoscale, detecting differences as small as 3 nm.

Out of focus images at a given *Z*-distance are stored and piled up. An image analysis algorithm detects differences between the congruent parts to finally produce in-focus images of very small particles at the level of nanoparticles, ten times smaller than the wavelength used.

#### 3.5.3. Diffuse Optical Tomography

The so-called “*photon-migration imaging*” is used to achieve a tissue penetration which exceeds OCT limits. This technique is used in *DOT or Diffuse optical tomography.*

It is based on the so-called tissue “spectral window” from 700 to 900 nm where light absorption due to chromophores, such as blood and water, is low. In this window, light diffuses due to high scattering, and this diffusion can be modelled. The main marker is blood. NIR (Near-IR) allows clear visualization of the oxygenation of haemoglobin and the quantity of circulating blood in muscle, cerebrum, or breast, for example. It is a **functional-NIR** showing blood response to tissue function. 

Other types of element detection require specific **molecular beacons** such as *tricarbocianine*, which is fluorescent to NIR, linked to a specific tumour protein, therefore showing the location of the tumour. It has also been used with other biomarkers such as amyloidal-b in Alzheimer's disease.

## 4. Discussion

Some years ago pathology departments were actively incorporating clinical specialists for specific organ biopsies (hepatic, renal, gynaecological, ophthalmologic, dermatologic, etc.). 

Now clinical departments will carry out morphological diagnoses based on noninvasive biopsy techniques. 

And pathologists will need to update their knowledge to cooperate at a distance (telediagnosis) otherwise laboratories will be empty of classical surgical diagnostic contents and full of advanced subspecialties according to new technology and technological advances [2]. 

We can conclude that nonstructured and structured illumination and interferometry, including holography and hybrid methods, produce high quality images comparable and sometimes better than optical microscopy and should be interpreted by a pathologist. This should be the **Optical Biopsy** section in pathology departments where optical biopsies are received in teleconsultation. Since OBs are taken by microendoscopic techniques, it is not feasible for pathologists always to be present, but their expertise is required to make an exact diagnosis. 

From the methods reviewed here, confocal endoscopy, PAM, and UH-OCT with or without contrast provide enough histological quality to be incorporated in diagnostic procedures, in collaboration with the pathology department, but for this, gold standards have to be established. 

Pathology knowledge and the gold standards of surgical pathology are built on the following premises: (1) Experience is better than evidence; (2) Knowledge based on the literature; (3) Scientific relevance or eminence; (4) Interpretation; (5) Personal impression. It seems obvious that the sooner we gain sufficient experience and images available and accessed by all surgical pathologists, the better and sooner the gold standard for optical biopsy will be established. OBs, particularly morphologically based, will only be incorporated into routine diagnostic procedures when its gold standards have been established. This is the reason why an image bank of optical biopsies accessible by the scientific community in a GRID environment is urgently needed, something similar to the protein atlas initiative [30]. Furthermore, we cannot forget the extreme efficiency of these techniques in medical support for underserved and isolated areas. 

We have seen the high microscopic and immunologic quality of confocal endomicroscopy, whose drawback is its limited tissue penetration (see Figure 1), which is why it is widely implemented in intestinal endoscopy. Alternative techniques such as PAM or UH-OCT with better penetration should progressively be introduced to widen the optical biopsy area. 

Furthermore the HOF technique can provide superior microscopic quality as compared to regular optical microscopy, and improvement of molecular techniques will extend immune-endoscopy towards pharmacy-pathology and personalized therapies as mentioned elsewhere [2].
